# Bilateral Vocal Fold Paralysis After Epidural Anesthesia

**DOI:** 10.7759/cureus.4212

**Published:** 2019-03-09

**Authors:** Jeffrey Saeks, Samuel B Reynolds, Jonathan S Alexander, Marion Ridley

**Affiliations:** 1 Otolaryngology, University of South Florida, Tampa, USA; 2 Internal Medicine, University of Louisville School of Medicine, Louisville, USA

**Keywords:** anesthesia, dysphagia, electromyogram, epidural, intrathecal

## Abstract

Cranial neuropathies are known potential complications of spinal anesthesia, with most reports describing upper cranial nerve involvement. Intrathecal hypotension resulting in traction injury of the cranial nerves is the likely mechanism of injury. Unilateral vagal neuropathy was first described recently. The patient discussed in this case presented with hoarseness and dysphagia after receiving epidural anesthesia for childbirth. Following videostroboscopy and laryngeal electromyogram, she was diagnosed with bilateral vocal fold paralysis. The patient was managed conservatively with expectant management. She exhibited complete spontaneous recovery, as has been the natural history previously described for similar injuries. The proposed mechanism for this patient, and in others described in the literature, is puncture of the dura with subsequent egress of cerebrospinal fluid, leading to intracranial hypotension and traction on cranial nerves. Unilateral vocal fold paralysis following spinal anesthesia has been reported in one case series consisting of three patients, but this represents the first case of bilateral paralysis. Spontaneous resolution has been observed in all patients. Patients presenting with idiopathic vocal fold paralysis, in summary, should be questioned about recent history of epidural or spinal anesthesia, as a positive history may point to transient intrathecal hypotension as a potential etiology of the paralysis.

## Introduction

Cranial neuropathies are known potential complications of spinal anesthesia. Most reports have described the involvement of the upper cranial nerves, meaning I and III to VIII [1-6]. Unilateral vagal neuropathy, signifying involvement of a lower cranial nerve (or IX to XII), following spinal anesthesia was first described recently [7-8]. Herein, a case of bilateral vocal fold paralysis after epidural anesthesia for childbirth was presented.

## Case presentation

The patient was a 37-year-old female who developed sudden breathy hoarseness and dysphagia two weeks after childbirth, with an associated headache that persisted for 14 days. An initial otolaryngologist evaluated the patient shortly after symptom onset and noted bilateral vocal fold paralysis on flexible laryngoscopy. Subsequent imaging included a computed tomography (CT) scan of the neck and chest as well as a magnetic resonance image (MRI) of the brain, both of which were unremarkable. A trial of antibiotics, which was attempted because of concern for pharyngitis, was ineffective. She was also offered steroids; however, she declined to take them for fear of breast milk transmission. She was then referred to a dedicated laryngologist as well as neurologist for further work up. 

The patient was evaluated by another otolaryngologist, who, on flexible laryngoscopy, noted bilateral vocal fold paralysis with the vocal folds fixed in the intermediate position as well as a mild palatal weakness indicative of a high vagal injury. Videostroboscopy three months after symptoms showed continued bilateral vocal fold fixation in the intermediate position but some slight movement of the arytenoids towards midline with maximal effort. Laryngeal electromyogram performed four months after symptoms showed evidence of severe denervation of the left thyroarytenoid muscle, neurogenic injury of the right thyroarytenoid muscle with evidence of reinnervation, and mild injury of the left cricothyroid with normal signals from the right cricothyroid (Table 1).

**Table 1:** Laryngeal needle electromyogram findings L = left, R = right, Fibs = fibrillations, PSW = positive sharp waves, Fasc = fasciculations, IP = interference pattern, sl. incr. = slow increase, MUP = motor unit potential. The columns "Fibrillations, Positive sharp waves, and Fasciculations" correspond to the spontaneous activity of the vocal folds. "--" indicate that no measurement was recorded for that value. The columns "Effort, Recruit, Interference pattern, Duration, Amplitude, and Complex" correspond to volitional motor unit action potentials observed during laryngeal needle electromyogram.

Muscle	Insertional activity	Fibs	PSW	Fasc	Effort	Recruit	IP	Duration	Amplitude	Complex
L Thyroarytenoid	Increased	3+	3+	None	Normal	--	--	No MUP	No MUP	--
R Thyroarytenoid	Normal	0	0	None	Normal	decreased	decreased	sl. incr.	sl. incr.	increased
L Cricothyroid	Normal	0	0	None	Normal	normal	normal	sl. incr.	sl. incr.	increased
R Cricothyroid	Normal	0	0	None	Normal	normal	normal	normal	normal	none

Repeat flexible laryngoscopy showed abduction and adduction of the right vocal fold but no movement on the left. Follow up exam six months after symptoms showed a normal function of the right vocal fold and near-normal function on the left. At this time, she noted that her voice was almost at baseline. At final follow-up nine months after symptoms, she had a complete return of bilateral vocal fold function and voice improvement back to her baseline.

## Discussion

Cranial neuropathies following dural puncture are a well-described transient phenomenon in the literature [1-6,8]. Most of the previously described reports involve upper cranial nerves. Abducens neuropathy, the most commonly reported neuropathy, manifests as diplopia on lateral gaze [1-5]. Oculomotor and trochlear neuropathies have also been described with symptoms of iridoplegia and diplopia [2,5]. Facial and trigeminal neuropathy have been reported leading to facial paralysis and diminished facial and tongue sensation [6]. Until recently, lower cranial neuropathies had yet to be reported. A 2014 case series, however, described three patients in whom unilateral vocal fold paralysis was noted up to one week after childbirth with spinal anesthesia as well as orthopedic procedures utilizing spinal anesthesia, with a proposed mechanism of vagal neuropathy [8]. And while this mechanism is unique, bilateral paralysis has not been described in the available literature, making this the first report of bilateral vocal fold paralysis after epidural anesthesia. 

A proposed pathophysiology of cranial neuropathies as a result of dural puncture has previously been described in the available literature. The consensus is that spinal dural puncture leads to an egress of cerebrospinal fluid, which can last for days. As a result, there is transient intrathecal hypotension leading to downward traction injury of the cranial nerves at the level of the brainstem [1-7]. It is important for providers to be aware that the potential for permanent nerve injury exists in such cases, and to exercise safety precautions in the course of administering epidural anesthesia accordingly.

The patient in the presented case underwent placement of an epidural catheter to administer anesthesia for vaginal childbirth. Under ideal circumstances, the placement of an epidural catheter should not cause any leakage of cerebrospinal fluid as there is no dural penetration involved in the procedure. However, an accidental dural puncture is a well-known complication during placement of epidural catheters and is believed to occur in 0.4% to 2% of cases [9-10]. In these cases headache is the most common symptom, occurring 80% of the time. The presented patient almost certainly had an accidental dural puncture and prolonged egress of cerebrospinal fluid as evidenced by her persistent headache. Also, her pattern of injury is characteristic of a high vagal insult as evidenced by her electromyogram findings of varying degrees of neurogenic injury in both the thyroarytenoids, innervated by the recurrent laryngeal nerve, as well as the cricothyroid innervated by the superior laryngeal nerve (Table 1). Her palatal weakness provides further support of a high vagal injury. Taken together, her symptoms of dural puncture as well as signs of high vagal injury support the hypothesis of traction injury on the vagus nerves bilaterally.

As is the case with other cranial neuropathies that occur after dural puncture, in which symptom resolution or at least substantial clinical improvement is seen in six months to one year, the presented patient had spontaneous resolution of her injuries over a period of nine months. On this note, the patient was not referred to speech therapy, as her symptoms resolved with supportive care alone, likely secondary to the restoration of circulation cerebrospinal fluid. While bilateral vocal fold paralysis has the potential to cause critical airway compromise, the discussed patient was found to have vocal fold fixation in the intermediate position [11]. This position allowed her to have an adequate airway and be managed expectantly without the need for tracheostomy. Due to the transient nature of these injuries, it is important to counsel patients on natural history and anticipated spontaneous recovery as part of their management. Since vagal neuropathy following dural puncture has only recently been described, injury to the dura with resultant intrathecal hypotension should be considered in patients with newly diagnosed bilateral vocal fold paralysis, specifically in patients who have received epidural spinal anesthesia.

## Conclusions

The diagnostic workup of idiopathic vocal fold paralysis should include questioning for a history of spinal epidural anesthesia, including for women in the postpartum period, as intrathecal hypotension can explain this presentation. Expectant management is the mainstay of therapy.
